# Differentiation-inducing therapeutic effect of Notch inhibition in reversing malignant transformation of liver normal stem cells via MET

**DOI:** 10.18632/oncotarget.24421

**Published:** 2018-02-05

**Authors:** Hao Luo, Wei-Hui Liu, Hong-Yin Liang, Hong-Tao Yan, Ning Lin, Dong-Yu Li, Tao Wang, Li-Jun Tang

**Affiliations:** ^1^ Third Military Medical University, Chongqing 400038, China; ^2^ General Surgery Center, Chengdu Military General Hospital, Chengdu 610083, China; ^3^ Department of Clinical Nutrition, Chengdu Military General Hospital, Chengdu 610083, China

**Keywords:** liver cancer stem cells, liver normal stem cells, malignant transformation, Notch inhibition, differentiation inducing therapy

## Abstract

**Background:**

Liver cancer stem cells (LCSCs) are the key factors for cancer metastasis, recurrent, and drug resistance. LCSCs are originated from either hepatocytes dedifferentiation or differentiation arresting of liver normal stem cells (LNSCs). Differentiation-inducing therapy is a novel strategy in solid tumors. Furthermore, Notch signaling pathway has been proved to play important role in the process of hepatocytes differentiation. In previous study, a malignant transformation cellular model of LNSCs has been built up, and in this study we are trying to illustrate whether inhibition of Notch can reverse this malignant tendency and drive these malignant cells back to differentiate into mature hepatocytes.

**Results:**

Inhibition of Notch signaling pathway can down-regulate the stemness-related cancer markers, lower the proliferative status, alleviate the invasive characteristic, or attenuate the metastasis tendency. What is more, it can help the malignantly transformed cells to regain the mature hepatic function of glucagon synthesis, urea metabolism, albumin production, and indocyanine-green (ICG) clearance.

**Materials and Methods:**

HOX transcript antisense RNA (HOTAIR) expression was enhanced in LNSCs via lentivirus transduction to set up the malignant transformation cellular model. Then, a Notch inhibitor was applied to induce malignantly transformed cells differentiate into mature hepatocytes, and malignant abilities of proliferation, invasiveness, tumorigenesis as well as mature hepatocyte function were observed and compared.

**Conclusions:**

The data demonstrate that the anti-tumor effects of Notch inhibition may lie not only on killing the cancer cells or LCSCs directly, it can also induce the LCSCs differentiation into mature hepatocytes via mesenchymal-epithelial transition (MET) progress or downgrade the malignancy.

## INTRODUCTION

Hepatocellular carcinoma (HCC) is one of the most common malignant tumors, with high progressive, invasive, and recurrent rate, as well as drug resistance. The theory of liver cancer stem cells (LCSCs) gives a reasonable explanation of these phenomena. The origination of LCSCs is still debated. It is believed that LCSCs originate from dedifferentiation of mature hepatocytes or differentiation arrest of liver normal stem cells (LNSCs). No matter which theory is more coincident with what happened *in vivo*, differentiation-inducing therapy can be an adoptable option targeted both dedifferentiation of mature hepatocyte and differentiation arrested of LNSCs.

Differentiation-inducing therapeutic effect of all-trans retinoic acid serves as a treatment for leukemia at first. However, with the recognition of cancer stem cells increasing, differentiation-inducing therapy is widely used in solid tumor such as glioblastomas, skin cancer, and lung cancer [1–3]. Meanwhile, many regimens which are used in leukemia such as all-trans retinoic acid has presented a differentiation inducing the therapeutic effect in hepatocarcinoma, as well as other medicine [4–7]. What is more, using hepatic corresponding differentiation-determining transcription factor such as hepatocyte nuclear factor (HNF) families, which are absent in HCC, can induce hepatoma cells differentiation into mature hepatocytes [8]. Differentiation inducing therapy can reverse the malignant phenotypes, prevent LCSCs from progressing and metastasis, and promote normal hepatic function recovery.

Notch signaling pathway has been proved to play a crucial role in both hepatocytes differentiation and anti-carcinogenesis. Notch signaling pathway can control the differentiation balance between hepatocytes and cholangiocytes [9]. In the previous studies, we find that inhibition of Notch signaling pathway acts as a hepatic differential effect of LNSCs [10, 11]. Meanwhile, Kang *et al* report that Notch activation drives adipocyte dedifferentiation and tumorigenic transformation in mice [12], while Notch inhibition presents multiple antitumor effect such as enhancing the sensitivity of chemo-therapy, suppressed cell growth, inducing apoptosis, and increasing cellular death [13–15]. To sum up, there are two possibly mehcanisms behind these antitumor effect. The first one is that inhibition of Notch is able to kill the HCCs directly. The other is that Notch inhibition plays the anti-tumor effect undirectly via inducing liver cancer cells differentiation into mature hepatocytes. However, whether Notch signaling pathway inhibition can demonstrate the differentiation inducing effect in malignantly transformed LNSCs is still unknown. Herein, we are trying to illustrate the differentiation-inducing therapeutic role of Notch signaling pathway inhibition in reversing the malignant transformation of LNSCs and regaining the lost hepatic function, and additionally to find out the potential mechanism.

## RESULTS

### Notch inhibition could reverses the high proliferative status of malignantly transformed LNSCs

**Figure 1:**
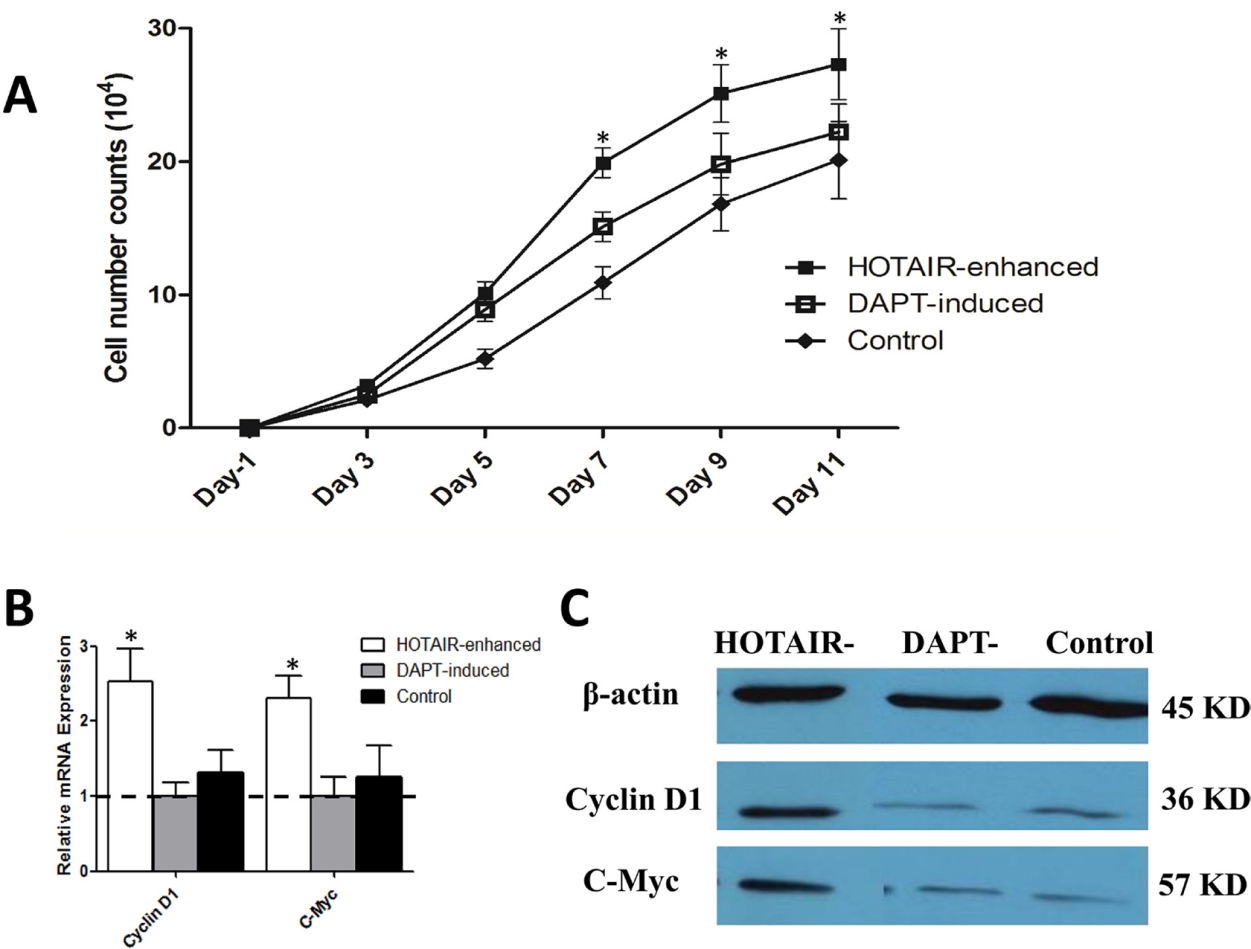
Notch inhibition could reverse the high proliferative status of malignantly transformed LNSCs (**A**) MTT assays showed that proliferative speed was similar in three groups at the early stage. From the 4th day, the number of cells in HOTAIR enhanced group was much higher than that the other two groups (*n =* 3, *P <* 0.05). Nevertheless, no significant difference was observed between DAPT-induced and control group (*n =* 3, *P >* 0.05). (**B**) At mRNA level, DAPT induced cells presented a lowest Cyclin D1 and c-Myc expression, and was set up as baseline. Cyclin D1 expression in HOTAIR enhanced group were 2.53-fold (2.53 ± 0.43, *n =* 3, *P <* 0.05) and 1.92-fold (1.92 ± 0.30, *n =* 3, *P >* 0.05) higher than DAPT induced and control group respectively. As to C-Myc, the xpression rate is 2.31-fold (2.31 ± 0.30, *n =* 3, *P <* 0.05) and 1.83-fold (1.83 ± 0.41, *n =* 3, *P >* 0.05). (**C**) WB results showed that the cellular cycle gene Cyclin D1 and proliferative gene c-Myc were downregulated in DAPT-induction or under-expressed in normal cells spontaneously (*n =* 3, *P <* 0.05) at protein level.

To exclude the proliferative status of cells in each group, cell proliferation was assessed by MTT and cellular cycle protein was detected by western-blot. During the timeline of the cell growth assay, the numbers of cells in each group were similar in the first 3 day (*n =* 3, *P >* 0.05). However, HOTAIR enhanced LNSCs were associated with a highest proliferative status after 3 days incubation (*n =* 3, *P <* 0.05). Meanwhile, DAPT-induction could slow down accelerated proliferative speed of HOTAIR-enhanced cells (*n =* 3, *P <* 0.05). What is more, both DAPT-induced and control cells presented a similar proliferative status, and the differences were not significant (*n =* 3, *P >* 0.05) (Figure 1A).

WB results showed that the cellular cycle gene Cyclin D1 and proliferative gene c-Myc, also known as an oncogene, would be downregulated in DAPT-induction or under-expressed in normal cells spontaneously (*n =* 3, *P <* 0.05) (Figure 1B and 1C). It indicated that DAPT could lead these uncontrolled cells back to normal proliferative status.

### Notch inhibition neutralizes the high invasive status of malignantly transformed LNSCs

**Figure 2:**
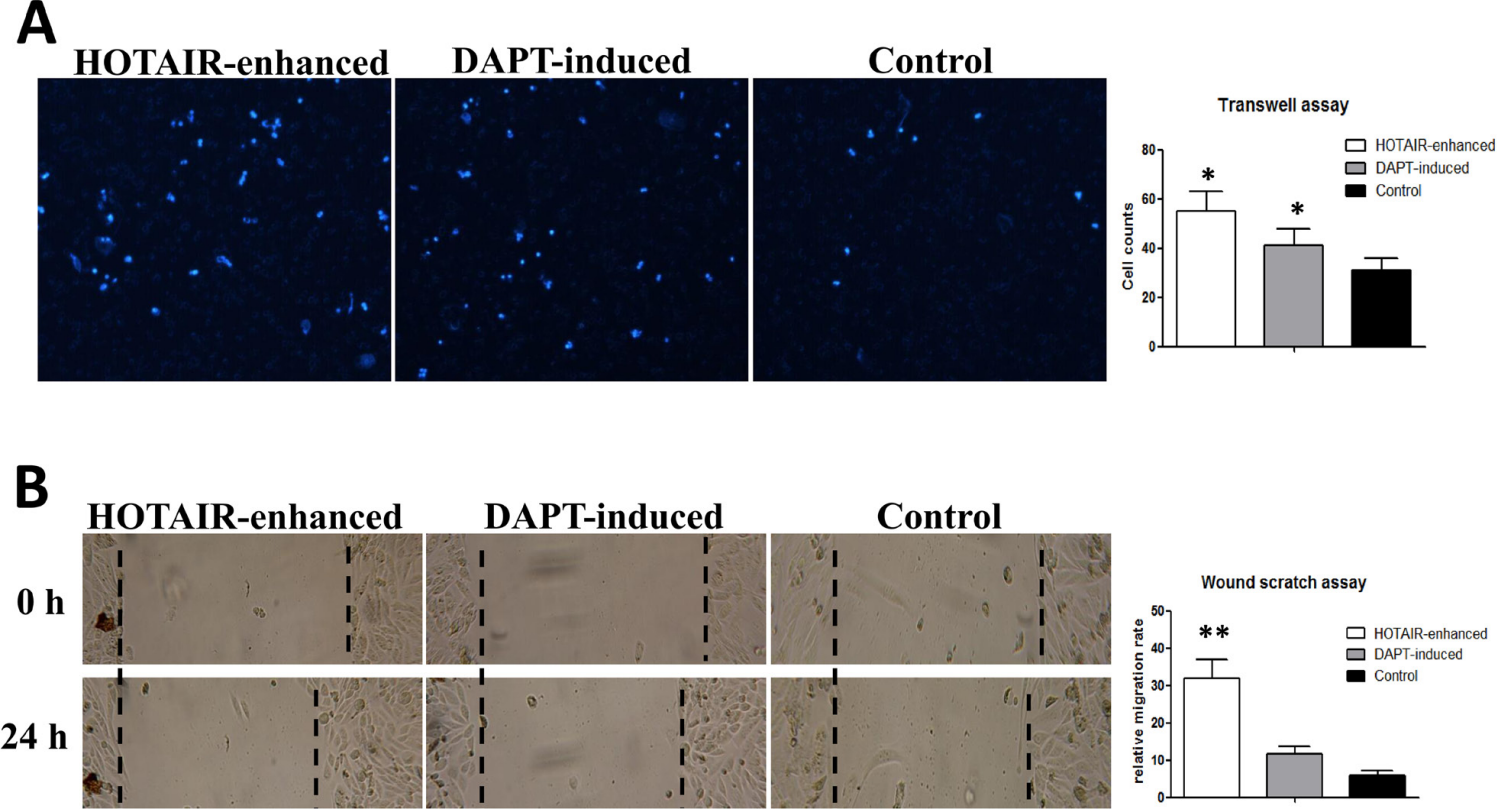
Notch inhibition neutralizes the high invasive status of malignantly transformed LNSCs (**A**) Transwell assays showed that the number of trespassed cells in HOTAIR enhanced group was 55/PHF (55 ± 8, *n =* 3, *P <* 0.05), 41/PHF (41 ± 7, *n =* 3, *P <* 0.05) in DAPT induced group, and 31/PHF (31 ± 5) in control group. (**B**) Scratch wound healing assays indicated that the gained migration ability of HOTAIR-enhanced cells could be absolutely counteracted by DAPT inducing (*n =* 3, *P <* 0.001).

To address the change of cellular invasion and migration, we performed transwell assays and scratch wound healing assays. Similar as the result of a previous study, we found it that HOTAIR-enhance could make the LNSCs gain a higher invasion and migration ability. Although more DAPT-induced cells trespass through the ECM than LNSCs, these trespassed cells were significantly less than that HOTAIR-enhanced group (*n =* 3, *P <* 0.05) (Figure 2A). It is demonstrated DAPT-induction could partly neutralize the invasion ability of malignantly transformed cells. However, through the scratch wound healing assays, the gained migration ability of HOTAIR-enhanced cells could be absolutely counteracted by DAPT inducing (*n =* 3, *P <* 0.001) (Figure 2B).

### Notch inhibition drives malignantly transformed LNSCs into mature hepatocytes

**Figure 3:**
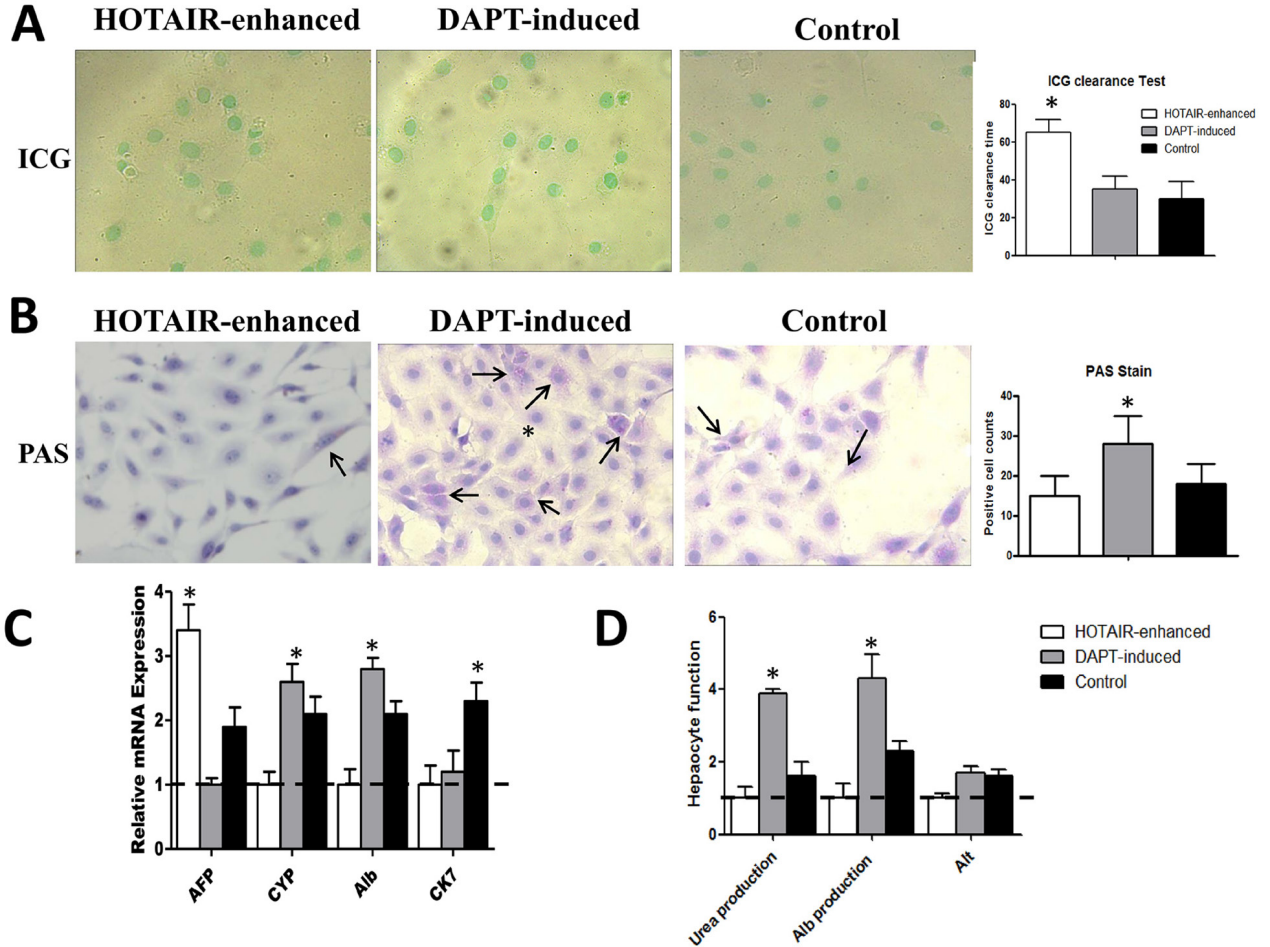
Notch inhibition drives malignantly transformed LNSCs into mature hepatocytes (**A**) In all three groups, cells were able to intake the ICG dye after incubation. However, the ICG clearance time is different in each group. In HOTAIR enhanced group, the clearance time is 65 minutes (65 ± 7, *n =* 3, *P <* 0.05), 35 minutes (35 ± 7, *n =* 3, *P >* 0.05) in DAPT-induced group and 30 minutes (30 ± 9) in control group. The difference between DAPT-induced group and control group was not significant. (**B**) The number of PAS-positive cells was 28/HPF (28 ± 7, *n =* 10, *P <* 0.05) in DAPT-induced group, and it is significantly higher than 15/HPF (15 ± 5, *n =* 10, *P >* 0.05) in HOTAIR enhanced group and 18/HPF (18 ± 5) in control group (*n =* 10, *P <* 0.001). (**C**) The lowest expression sample was set up as baseline. Immature hepatic marker AFP was 3.3-fold (3.3 ± 0.4, *n =* 3, *P <* 0.05) and 1.8-fold (1.8 ± 0.3, *n =* 3, *P >* 0.05) higher than that in DAPT-induction and LNSCs respectively. CK-7 expression was highest expressed in LNSCs (*n =* 3, *P <* 0.05). The patterns of mature hepatic marker, CYP and Alb expression were similar. DAPT-induced cells presents a highest expression of these mature hepatic markers, while HOTAIR enhanced cells displayed a lowest expression of mature hepatic markers (*n =* 3, *P <* 0.05). (**D**) As to as for Urea production, Alb production and Alt production, DAPT-induced cells exhibit a significantly higher expression than the other two groups (*n =* 3, *P <* 0.05). Black arrow showed the PAS-positive cells.

To address whether DAPT-induction promotes malignantly transformed LNSCs differentiation into mature hepatocytes, the excretory, synthetic, and metabolic function were evaluated. ICG was broadly used to evaluate the liver function. Herein, it was found that all three kinds of cells could intake ICG to the cytoplasm (Figure 3A). The difference was that HOTAIR-enhanced cells were able to absolutely eliminate the dye after about 1 hour. Quite differently, DAPT-induction could significantly shorten the elimination time to the level about 30 minutes, which was similar with normal LNSCs (*n =* 3, *P <* 0.05) (Figure 3A).

The PAS staining was used to illustrate the change of synthetic function. the number of PAS-positive cells in HOTAIR group was 14/HPF, 29/HPF in DAPT-induction group (*n =* 10, *P <* 0.05), and 16/HPF in LNSCs respectively (Figure 3B).

To demonstrate the difference of metabolic function of each group, the immature hepatic marker AFP, immature cholangiocytes marker CK7, and mature hepatic marker CYP as well as Alb were calculated using qRT-PCR. The Urea production, Alb production and Alt production were analyzed using an automatic biochemical analyzer. The lowest expression sample was set up as baseline. Immature hepatic marker AFP was 3.3-fold and 1.8-fold higher than that in DAPT-induction and LNSCs respectively (*n =* 3, *P <* 0.05). Because LNSCs were bipotential cells which presents hepatic markers as well as cholangiocytic markers, CK-7 expression was highest expressed in LNSCs (*n =* 3, *P <* 0.05). The patterns of mature hepatic marker, CYP and Alb expression were similar. DAPT-induced cells presents a highest expression of these mature hepatic markers, while HOTAIR enhanced cells displayed a lowest expression of mature hepatic markers (*n =* 3, *P <* 0.05) (Figure 3C). As to as for Urea production, Alb production and Alt production, DAPT-induced cells exhibit a significantly higher expression than the other two groups (*n =* 3, *P <* 0.05) (Figure 3D)

### Notch inhibition alleviates the carcinogenesis tendency of malignantly transformed LNSCs *in vivo*

**Figure 4:**
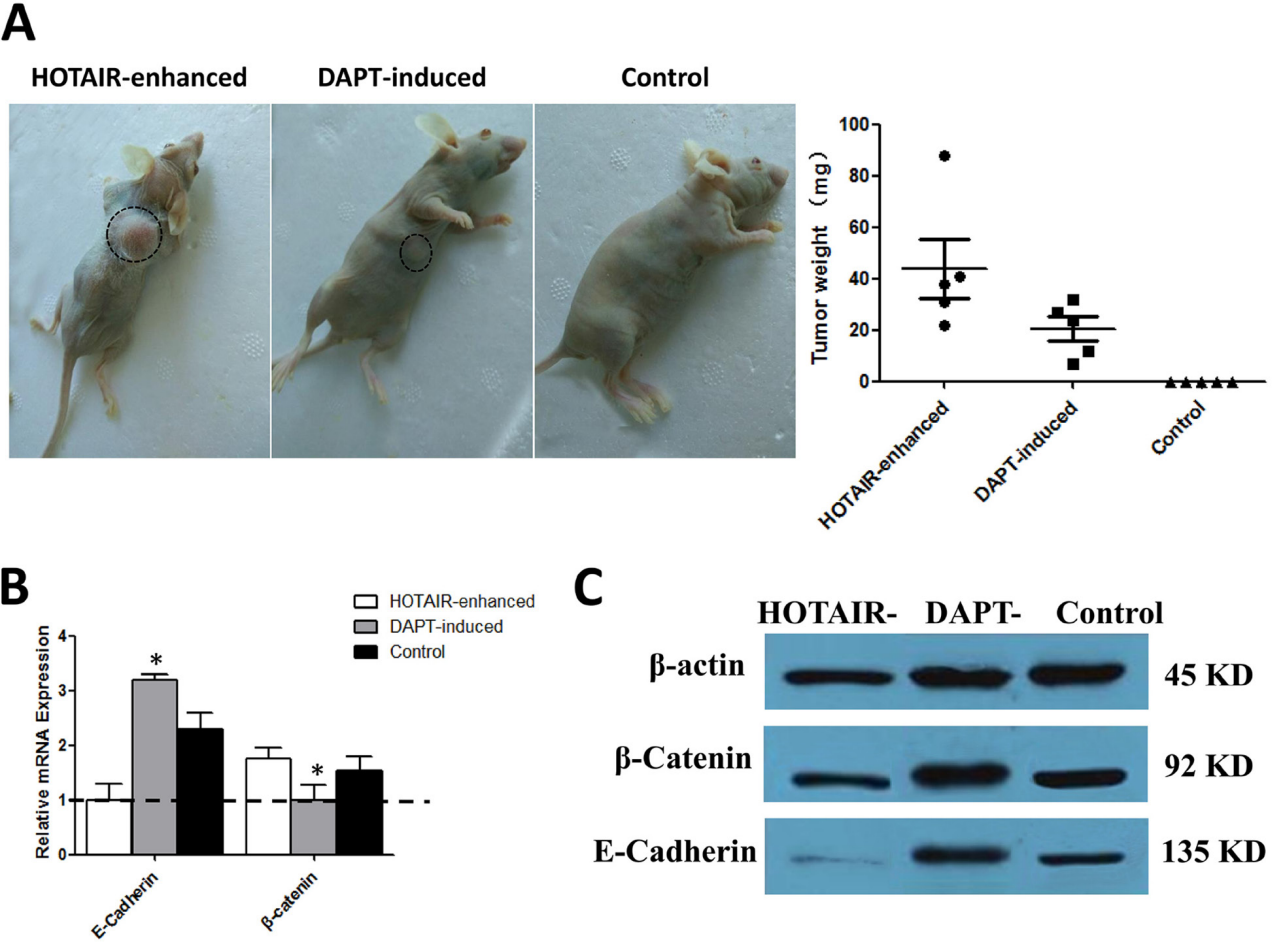
Notch inhibition alleviates the carcinogenesis tendency of malignantly transformed LNSCs via EMT (**A**) The results showed that HOTAIR-enhanced cells developed visible tumors on every nude mouse, whereas no visible tumor was observed on the mice which were injected with LNSCs. Meanwhile, the cells handled with DAPT-inducing formed a much smaller tumor than that formed in HOTAIR-enhanced cells. (**B**, **C**) The results suggested that the MET markers E-Cadherin expression was up-regulated not only at the mRNA level (3.2-fold higher than baseline, 3.2 ± 0.1, *n =* 3, *P <* 0.05) but also at the protein level in DAPT-induced cells. Oppositely, EMT marker β-catenin was up-regulated at both mRNA level (1.8-fold higher than baseline, 1.8 ± 0.3, *n =* 3, *P <* 0.05) and protein level in HOTAIR-enhanced cells. The black cycle indicated the neoplastic tumor. The lowest expression sample was set up as baseline.

In previous assays, the *in vitro* tumor characteristic changes of proliferation, invasive, migration, cell markers, and hepatic function were tested. Tumorigenesis in immune-deficient mice was recommended as the ‘‘smoking gun’’ for cancer formation and for evaluation of anti-tumor therapy. To test the tumorigenic ability of cells from each group, equal numbers of cells were injected sub-percutaneously into mice. The results showed that HOTAIR-enhanced cells developed visible tumors on every nude mouse, whereas no visible tumor was observed on the mice which was injected with LNSCs. Meanwhile, the cells handled with DAPT-inducing formed a much smaller tumor than that formed in HOTAIR-enhanced cells (Figure 4A).

### The molecular mechanism of Notch inhibition in differentiation inducing therapy

To explore the molecular mechanism behind the differentiation inducing therapy, the EMT markers E-cadherin and β-catenin was detected by WB. The results suggested that MET marker E-cadherin expression was up-regulated not only at the mRNA level (Figure 4C) but also at the protein level (Figure 4B) in DAPT-induced cells. Oppositely, EMT marker β-catenin was up-regulated at both mRNA level (Figure 4C) and protein level (Figure 4B) in HOT AIR-enhanced cells. HOTAIR enhancing can lead to E-cadherin degraded and β-catenin up-regulated, which resulted in EMT, while DAPT differentiation inducing therapy could reverse this tendency and trigger the MET processing.

## DISCUSSION

Where and how LCSCs derive from is the core problem of HCC therapy. It is believed that LCSCs originate from dedifferentiation of mature hepatocytes or differentiation arrest of liver normal stem cells (LNSCs) with many molecules involved in such as Hippo and Notch signaling pathway. Mu *et al.* found that AFP-, A6-, and CK19-positive cells within HCCs may be hepatocyte-derived cells that underwent dedifferentiation, and the progenitor signature of HCCs does not reflect progenitor origin, but dedifferentiation of hepatocyte-derived tumor cells [16]. Under specific conditions, hepatocytes present multi-plasticity and dedifferentiate into biliary cells and even immature progenitors. This dedifferentiation ability results in the presentation of an advantage for the tumor [17]. Another finding indicates that loss of Hippo-pathway signaling and upregulation of YAP drive the mature hepatocytes to dedifferentiate into progenitor characteristics [18]; what is more, transgenic mice with YAP enhanced results in increased hepatocyte dedifferentiation, cell migration, EMT, and malignant transformation of LNSCs [19]. It is of great interest that the dedifferentiation of HCCs may predict a worse prognosis [20]. Meanwhile, a quite different idea believes that LCSCs arise out of differentiation and/or maturation arrest of LNSCs [21]. Chiba *et al.* transferred Bmi1 and Wnt/beta-catenin into c-Kit^(-)^CD29^(+)^CD49f^(+/low)^CD45^(-)^ Ter-119^(–)^ hepatic stem/progenitor cells and these cells finally initiate hepatic tumors [22]. With P53 gene deleted in LNSCs, Suzuki *et al.* suggested that these transgenic cells can result in the formation of liver tumors [23]. In our previous study, suppressing the anti-oncogene Tg737 and miRNA-200a, or enhanced oncogene miRNA-10b and HOTAIR could lead LNSCs to malignant transformation by promoting the cell-cycle progressing and differentiation arrest respectively [24–26].

No matter which theory above is more applicable to the real pathologic procedure of HCC initiation, differentiation-inducing therapy can target both dedifferentiation and differentiation arrest. Although differentiation-inducing therapy has first been administrated in hematologic tumors, recent studies have applied this strategy to liver cancer. All-trans-retinoic acid is powerful weapon for leukemia. However in liver cancer, all-trans-retinoic acid can alleviate the dedifferentiation effect which is resulted from the upregulated expression of both TGF-β1 and CD147, and play a differentiation-inducing effect [27]. Zhang *et al.* demonstrated that all-trans-retinoic acid can not only contribute to differentiation-inducing therapy function, can also boost the chemo-sensitive of HCCs [4]. Xie *et al.* illustrated that the core differentiation factors HNF-1α and -4α were absent in HCC and compensative upregulation of these absent factors can induce CD133^(+)^ LCSCs differentiation into mature hepatocytes [8, 28]. Oncostatin M (OSM) is known to be able to induce the LNSCs into hepatocytes. However in the EpCAM^(+)^ stem phenotype HCCs, OSM can decrease the stemness-related gene expression, such as EpCAM, α-fetoprotein and CK19 [7]. Similar with OSM, in our previous study, it was demonstrated that Notch inhibition could promote FLSPCs differentiation into hepatocytes, and these differentiated cells presented multiple hepatic functions [10, 11]. It is true that differentiation-inducing therapy is novel therapeutic method for solid tumor such as HCC. We believe that the feasibility of differentiation-inducing therapy lies on several facts. First, many antitumor reagents, like miR-148a, DMSO and Notch signal inhibitor DAPT, are also known as differentiation inducer. Jung [29] identified miR-148a as an inducer of hepatic differentiation, while MiR-148a-mimetic treatment *in vivo* suppressed tumor growth, reduced tumor malignancy in liver. Dimethyl sulfoxide (DMSO) can enhance the chemo-sensitive response to chemotherapeutic agents in HCC [30], while DMSO can also accelerate the hepatogenic procedures of adipose tissue-derived mesenchymal stem cells [31]. Similar patterns happen to DAPT [32, 33]. In this study, we are trying to figure out whether inhibition of Notch can play differentiation-inducing therapy and reverse the malignant transformation procedures.

Although differentiation-inducing therapy will not kill the HCCs or LCSCs directly, it can down-regulate the stemness-related cancer markers, lower the proliferative status, alleviate the invasive characteristic, or attenuate the metastasis tendency. Previous studies focus on the fact that differentiation therapy can down-regulate cancer markers, while the fact that the hepatic function recover after differentiation therapy is the more important. In this study, we lay our emphasis not only on the cancer markers, but also on the recovery of the hepatocyte function. On cancer characteristic, inhibition Notch could decrease the proliferation through downregulation of oncogene c-Myc and Cyclin D1. It can reverse the invasive status, terminate metastasis tendency, and decrease tumorigenesis ability. More meaningfully, it will help the malignantly transformed cells to regain the mature hepatic function of glucagon synthesis and ICG elimination. Clearance of ICG is an important factor to evaluate the hepatic function. Additionally, ICG was found highly loaded at tumor tissue, and helped to identify the margin of the tumor [34]. Because the HCCs are not able to eliminate the ICG, it will accumulate in the tumor tissue; what is more, the accumulation of ICG is correlated with the differentiation status of HCCs [29, 30]. Well-differentiated HCCs show light and uniform fluorescence of ICG in the cancerous tissue, while poorly differentiated HCCs show strong and rim-type fluorescence around the tumor [35]. Our results demonstrated that Notch inhibition was able to accelerate the clearance speed of ICG in malignantly transformed cells due to the promotion of the differentiation status. It is a sign for both LNSCs differentiation into mature hepatocytes and LCSCs response to differentiation-inducing therapy.

**Figure 5:**
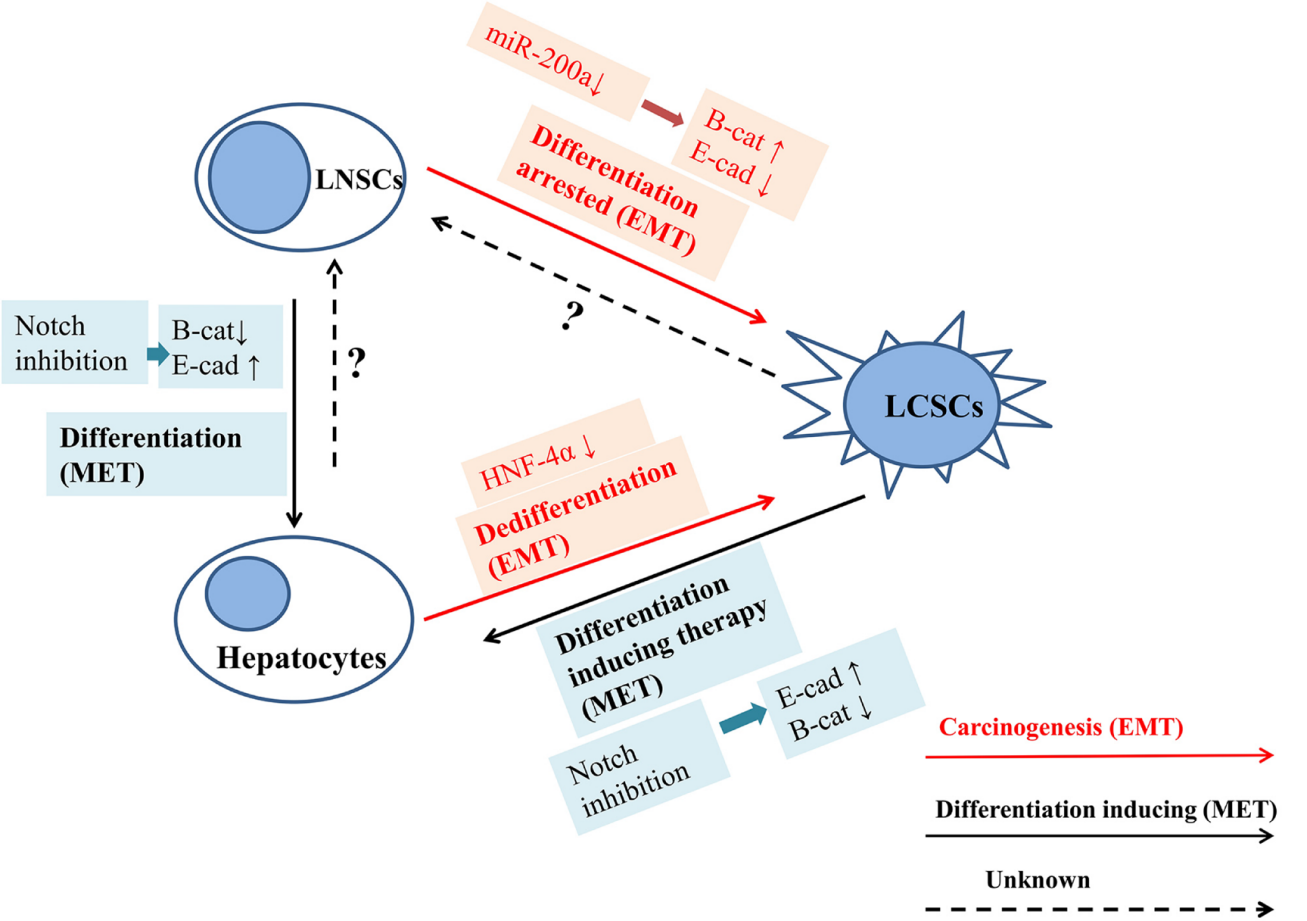
The mechanism of differentiation inducing therapy EMT process is involved in malignant transformation and drives the LNSCs into LCSCs via either dedifferentiation or differentiation arrested, while the MET process is the initiative factors for LNSCs maturation and differentiation. It has been proved that many molecules and signaling pathways are involved in such as HNF families, miRNA families, and Wnt signaling pathways. The anti-tumor effects of Notch inhibition may lie not only on killing the HCCs or LCSCs directly, it can also induce the LCSCs differentiation into mature hepatocytes via MET progress and downgrade the malignancy. The red arrow shows the process of EMT or malignant transformation, and the black arrow indicates the process of differentiation inducing therapy and MET.

The mechanisms behind the malignant transformation and differentiation-inducing therapy are complicated. Many evidences give clues that EMT process is involved in malignant transformation and drive the LNSCs into LCSCs via either dedifferentiation [36] or differentiation arrested (red arrow in Figure 5) [37, 38], while the MET process is the initiative factors for LNSCs maturation and differentiation (black arrow in Figure 5). Differentiation-inducing therapy is novel therapeutic strategy for solid tumor management. It has been proved that many molecules and signaling pathways are involved in such as HNF families, miRNA families, Wnt signaling pathways [39, 40]. In this study, we found that the anti-tumor effects of Notch inhibition may lie not only on killing the HCCs or LCSCs directly, it can also induce the LCSCs differentiation into mature hepatocytes via MET progress and downgrade the malignancy. However, there are still some unsolved paradoxes in differentiation-inducing therapy. For one thing, inhibition of Notch promotes LNSCs differentiate into hepatocytes, and arrest differentiation of cholangiocytes [41, 42], while Notch activation drives carcinogenesis, development and progression of cholangiocarcinoma in mice and human [43–45]. The paradoxical role of Notch in cholangiocytes differentiation and cholangio-carcinogenesis is our future research interests. For another, although it is sure that the destination of differentiation inducing therapy is mature hepatocytes, whether LNSC is a transition state between LCSC and mature hepatocyte, or differentiation inducing therapy can directly drive LCSCs to differentiate into mature hepatocytes it is still unknown.

## MATERIALS AND METHODS

### Cell isolation, culture and transfection

Adult experimental F344 rats were purchased from West China Medical School of China. The Research Animal Ethics Committee at the Chengdu Military General Hospital approved the animal study protocol (animal protocol number CMGH-2013-023).

The isolation and purification of LNSCs were performed as described previously [46]. The cells were grouped according to the transfection conditions as follows: HOTAIR enhanced group and HOTAIR enhanced plus Notch inhibition group (DAPT group, with a DAPT concentration of 1 μM). The cells in negative control group were treated with dimethyl sulfoxide (DMSO) (control group). Cells in each group were cultured in Williams’ E medium (Sigma, USA) with 15% (v/v) fetal bovine serum (Invitrogen, USA). The cells were incubated under 5% (v/v) CO_2_ at 37°C.

In HOTAIR enhanced group, pCDNA/HOTAIR was transfected into LNSCs using Lipofectamine 2000 according to the manufacturer’s instructions.

### MTT assay

The cellular proliferative ability from each group was determined through the 3-(4,5-dimethylthiazol-2-yl)-2,5-diphenyltetrazolium bromide (MTT) assay. The cells from each group were cultured in 96-well plates at a concentration of 5 × 10^3^ cells/well. Sterile PBS including 20 μl MTT (final concentration of 5 mg/ml) (Sigma, USA) was added to each well and incubated at room temperature for 4 h, and a continuous detection were made at 1, 3, 5, 7, 9, and 11 days of culture. To fully dissolve the crystal, 150 μl DMSO (Invitrogen, USA) was added into each well. The optical density was measured at a wavelength of 490 nm with a Bio-Rad 680 microplate reader (Bio-Rad, USA).

### Transwell migration assay

A total cellular number of 5 × 10^3^ in 500 μl of serum-free Williams’E medium from each group were seeded into an 8-μm pore-size polycarbonate membrane Boyden chamber, which was inserted into a transwell apparatus (Costar, MA, USA). Additional 500 μl Williams’ E medium containing higher FBS (15%) was added into the lower layer of 24-well plate. After incubated at 37°C for a day, the trespassed cells were fixed in 95% ethanol for 5 min, stained with DAPI (1 μg/mL), rinsed in PBS for 3 × 5 mins, and observed via fluorescence microscopy (Olympus, Japan).

### Scratch wound healing assays

A total number of 5 × 10^3^ cells from each group were added into 6-well plate and incubated overnight in FBS free medium. Pipette tips were used to make wounds on the attached growing cells. The wound healing procedure was observed for 24 h, and dead cells were gently washed off before photographs were taken.

### Real time polymerase chain reaction (RT-PCR)

RNA was harvested using Trizol (Invitrogen, USA), according to the manufacturer’s instructions. The cDNA synthetization was performed as Primescript RT reagent Kit (TAKARA, China). The specific primers were exhibited in Table 1. PCR reactions were performed as following: template denaturation at 95°C for 1 min, primer annealing at 60°C for 1 min, primer extension at 72°C for 2 min (35 cycles), and 72°C for 10 min as an extra cycle of elongation.

**Table 1:** Primers sequences for QRT-PCR

Name	Accession number	Primer sequences	Product size (bp)
AFP F	NM_012493.2	ctgta tgctc ccacc attat tt	109
AFP R		ttgat gctct ctttg tctgg aa	
ALB F	NM_134326.2	gacaa agcag cctgc ctgac	174
ALB R		ttctg cgaac tcagc attgg	
β-Catenin F	NM_053357.2	actcc aggaa tgaag gcgtg	109
β-Catenin R		gaact ggtca gctga accga	
CYP	NM_013105.2	cattc ctcac gccag tatat ga	198
CYP		cggat agggc tgtat gagat tc	
CK7 F	NM_001047870.1	aggaa cagaa gtcag ccaag ag	210
CK7 R		gcaac acaaa ctcat tctca gc	
C-Myc F	NM_012603.2	cgagc tgaag cgtag ctttt	170
C-Myc R		ctcgc cgttt cctca gtaag	
Cyclin-D1 F	NM_171992.4	gcgta ccctg acacc aatct	233
Cyclin-D1 R		ggctc cagag acaag aaacg	
Gapdh F	NM_017008.4	atggt ggtga agacg ccagt a	143
Gapdh R		ggcac agtca aggct gagat g	

### Protein extraction and western-blot (WB)

The cells were pyrolysis in RIPA buffer containing protease inhibitors. The lysates were centrifuged at 10,000 rpm for 10 min at 4°C. After centrifuge, the supernatant were collected. Total of 50 µg protein was uploaded to sodium dodecyl sulfate polyacrylamide gel electrophoresis and blotted onto a PVDF membrane. The membrane was blocked using 5% fat-free milk at room temperature for 2 h. The concentration of antibody for incubated is as following: anti-E-cadherin (1:200), anti-Cyclin D1 (1:1,000), anti-C-Myc (1:1,000), anti-β-catenin (1:500), anti-β-actin (1:1,000) (Sigma, USA) at 4°C for overnight. After three washes for 5-min in TBST, the membrane was incubated in peroxidase-conjugated goat anti-mouse/rabbit IgG or peroxidase-conjugated rabbit anti-goat IgG (Abcam, US) for 2 h at room temperature. An electrogenerated chemiluminescence system was used to visualize the proteins.

### Xenograft mouse model

About 5 × 10^7^ cells from each group were subcutaneously injected into the nude mice on the back separately. All mice were sacrificed 60 days after injection, and the weight of the tumor was detected.

### Statistical analysis

All of the experiments were independently replicated triply. Statistical analysis was performed using SPSS Version 19.0 (SPSS, USA). Continuous variables were evaluated using Fisher’s exact *t*-test or Mann-Whitney *u*-test. Discrete variables were assessed using the Chi-square test. Two-tailed *P* values < 0.05 were considered statistical significance.

## Abbreviations

AFP: alpha-fetoprotein
HPF: high power field
CT: cycle threshold
CYP: cytochrome P450
CK: cytokeratin
PCR: polymerase chain reaction
DAPT: N-[N-(3: 5-Difluorophenacetyl)-L-alanyl]-S-phenylglycine t-butyl ester
LCSCs: liver cancer stem cells
LNSCs: liver normal stem cells
ICG: indocyanine green
PAS: Periodic Acid Schiff’s staining
ncRNA: non-coding RNA
EMT: epithelial-mesenchymal transition
MET: mesenchymal-epithelial transition
HOTAIR: HOX transcript antisense RNA
HNF: hepatocyte nuclear factor
DMSO: dimethyl sulfoxide
